# Audit of Sleep Assessment and Management in Older Adults Across Mental Health Services: Impact of a Targeted Educational Intervention

**DOI:** 10.1192/bjo.2026.11555

**Published:** 2026-06-30

**Authors:** Gabriel Augusto Araujo Vieira Marcque, Muhammad Khalil, Ahmed Abdelsamie, Akhila Krishnan, Surya Goudaman

**Affiliations:** 1 Oxleas NHS Foundation Trust, London, United Kingdom; 2 South East London and Maudsley NHS Foundation Trust, London, United Kingdom

## Abstract

**Aims:**

To evaluate clinician knowledge, confidence, and practice in assessing and managing sleep problems in older adults within mental health services; to assess alignment with UK national guidance (NICE, NHS, RCPsych); and to evaluate the impact of a targeted educational intervention on sleep assessment, use of structured tools, and non-pharmacological management strategies.

**Methods:**

A quality improvement audit was conducted across older adult inpatient wards and Community Mental Health Teams within Oxleas NHS Foundation Trust, Bromley. The project used a pre- and post-intervention clinician survey alongside a pre-intervention review of patient records. Twenty clinicians completed the pre-intervention survey, with 11 completing post-intervention. Thirty patient records from inpatient and CMHT settings were reviewed at baseline. The audit evaluated clinician confidence, routine practice in sleep assessment, use of structured tools (sleep diaries, PSQI, Epworth Sleepiness Scale, STOP-BANG), awareness of national guidance (NICE NG97 and NG222), and quality of documentation and management strategies. The intervention comprised targeted teaching on sleep assessment in older adults, dissemination of guidance on non-pharmacological approaches, and emphasis on holistic assessment, age-related sleep physiology, and safer prescribing practices.

**Results:**

Before the intervention, 45–50% of clinicians reported routinely assessing sleep; 80% did not use structured tools, and only 10% used sleep diaries. Awareness of UK guidance was limited, with 80% unfamiliar. Patient record review showed 23.3% contained detailed assessments aligned with guidance, 70% brief mention only, and 6.7% had no documentation. Pharmacological management was frequent (88.2%), with limited non-pharmacological interventions.

After the intervention, 90% reported routine sleep assessment. Use of structured tools increased to 81%, with 72% using sleep diaries. Awareness of guidance improved (72% familiar), alongside increased confidence and greater consideration of age-related pharmacological risks (falls, sedation, confusion). Ninety percent reported routinely offering sleep hygiene advice.

**Conclusion:**

Sleep problems in older adults were under-assessed and inconsistently documented. A targeted educational intervention led to substantial improvements in clinician confidence, structured assessment, awareness of national guidance, and use of non-pharmacological strategies. Focused, low-cost education can significantly enhance the quality and safety of sleep assessment and management in older adult mental health services. Embedding regular training, structured tools, and clear guidance is recommended, with re-audit planned to assess sustainability.

